# Relationship between Hepatitis B Virus Infection and Celiac Disease

**Published:** 2010-12-01

**Authors:** Asma Ouakaa-Kchaou, Dalila Gargouri, Jamel Kharrat, Abdeljabbar Ghorbel

**Affiliations:** 1 Department of Gastroenterology and Hepatology, Habib Thameur Hospital, Tunis, Tunisia

**Keywords:** Hepatitis B virus infection, Celiac disease

Dear Editor,

We read with great interest the article entitled "are hepatitis B virus and celiac disease linked?" by Leonardi, et al, [1] published in Hepatitis Monthly. Celiac disease (CD), also known as gluten-sensitive enteropathy and non-tropical sprue, is a prevalent autoimmune disorder. The balance of evidence suggests that the celiac immunopathology involves a complex individualized interplay of many pathophysiological variables on a genetic background [2]. CD develops as a consequence of the encounter between an environmental trigger (i.e., derivatives of gluten from wheat, rye, and barley), immunologic factors, and a genetically predisposed host, with the possible participation of other environmental cofactors. In particular, intestinal infections might cause a transient rise in small-bowel permeability that could lead to up-regulation and release of tissue transglutaminase which in turn, enhances gluten immunogenicity. Rod-shaped bacteria have been identified in the intestinal epithelium in children with CD, although this colonization could just be coincidental [3]. Rotavirus infections could also raise the risk of CD in genetically predisposed children. The homology between the rotavirus-neutralizing protein VP-7 and tissue transglutaminase might explain how rotavirus infection is implicated in the development of CD [3]. It has also been hypothesized that hepatitis B virus (HBV) and hepatitis C virus (HCV) may trigger immunologic gluten intolerance [4][5]. Although the association between CD and several liver disorders has long been documented, no definitive evidence is available about the association between HBV or HCV and CD. The role of HCV infection is better documented and several studies have described the relationship between HCV infection and the development of CD [4]. However, little data exist on the relationship between HBV and CD and it seems that no such relation is found concerning HBV immunization. The main finding of the study of Leonardi [1] is the absence of any relationship between CD and HBV. However, the sample size was small, the studied group was heterogeneous including those with ongoing and resolved HBV infection, there was no control group, endoscopy and duodenal biopsy were not performed in patients who tested positive for either IgA or IgG anti-gliadin antibodies (Ig AGA) in whom symptoms of CD may be lacking. Activation of silent CD during the antiviral treatment with interferon-α has also been reported. Nevertheless, we agree with Leonardi, et al, that it is not mandatory to check for specific CD antibodies before beginning treatment with interferon and during follow-up.In conclusion, we suggest that the findings of this study should be open to a more conservative interpretation. Therefore, large follow-up studies, including a sample size that is more representative of the prevalence of CD, are needed to clarify how HBV infection may affect the development of this condition and to identify primary prevention strategies.
